# Implementing the Ideal Clinic Program at Selected Primary Healthcare Facilities in South Africa

**DOI:** 10.3390/ijerph18157762

**Published:** 2021-07-22

**Authors:** Livhuwani Muthelo, Faith Moradi, Thabo Arthur Phukubye, Masenyani Oupa Mbombi, Rambelani Nancy Malema, Linneth Nkateko Mabila

**Affiliations:** 1 Department of Nursing Science, University of Limpopo, Mankweng 0727, South Africa; moradifaith@gmail.com (F.M.); arthur.phukubye@ul.ac.za (T.A.P.); masenyani.mbombi@ul.ac.za (M.O.M.); 2 Department of Psychology, University of Limpopo, Mankweng 0727, South Africa; nancy.malema@ul.ac.za; 3 Department of Pharmacy, University of Limpopo, Mankweng 0727, South Africa; nkateko.mabila@ul.ac.za

**Keywords:** Ideal Clinic, infrastructure, professional nurses

## Abstract

Background: Primary healthcare (PHC) in South Africa often experiences crucial challenges that lead to patients’ negative experiences regarding their care, compromising the significant role that PHC services could play in health promotion and disease prevention. The primary purpose of implementing the Ideal Clinic (IC) in South Africa was to improve patients’ care quality at the clinics. There seems to be a paucity of studies determining professional nurses’ experiences when implementing the IC. Purpose: This study aimed to explore and describe professional nurses’ experiences regarding implementing the IC at three selected clinics in the Makhado local area. Study method: A qualitative phenomenological research design was used to explore professional nurses’ experiences regarding IC implementation. Purposive sampling was used to select 15 professional nurses working at the three selected clinics. Data were collected using semi-structured one-on-one interviews. Interviews were conducted until saturation was reached. Trustworthiness was ensured by applying Lincoln and Guba’s four criteria, i.e., credibility, transferability, dependability, and confirmability. Ethical clearance was obtained from the University of Limpopo Turfloop Research and Ethics Committee, and permission to conduct the study was obtained from Limpopo Province Department of Health Research and Ethics Committee. Thematic analysis was used to analyze data. Results: The following themes emerged from the study findings: perceived benefits of the IC on the primary healthcare services provided to the community, challenges experienced by professional nurses when implementing the IC program, and challenges related to the supply of resources for implementing the IC. The study results revealed that, although the IC aimed to improve the overburdened PHC facilities in SA, the professional nurses still experienced some challenges when implementing the IC program. Some of the challenges faced were a lack of knowledge and training in the IC program, poor infrastructure and the shortage of equipment, and inadequate provision of support by line managers, all of which resulted in poor-quality patient care. Conclusion: This study revealed that the introduction and implementation of the IC can have potential benefits to the community and the primary healthcare system. However, it was not introduced and appropriately implemented, which resulted in professional nurses experiencing several challenges. The national department of health needs to strengthen the program’s implementation through proper training, consultation, and continuous support of the nurses. Provision of quality equipment and supplies is also recommended.

## 1. Introduction

According to the 2018 global joint report by the World Health Organization (WHO), Organization for Economic Cooperation and Development (OECD), and World Bank Group, globally, poor-quality health services are holding back progress on improving health in countries at all income levels [1,2,3]. Additionally, in 2018, different countries in the world reached an agreement to strengthen the primary healthcare (PHC) services to achieve universal healthcare for all [4]. Universal healthcare coverage is considered the main objective of the Sustainable Development Goals, which should be achieved by 2030 [5]. However, very few countries took the initiative to improve the quality of healthcare services. Malaysia is one of the countries in Asia which took the initiative, wherein they used the “Operation Phakisa” method, a term in Sesotho meaning “hurry up.” South Africa adopted this method to implement an IC program from the Malaysia significant fast results approach [6]. The Malaysian approach aimed to achieve rapid transformation within a short time to address national priorities such as poverty, crime, and unemployment. The approach included laboratory planning, ensuring the availability of medicine and supplies, as well as creating healthcare worker training to meet the requirements to provide professional, efficient, effective, and sustainable delivery of healthcare services [6].

Different scholars have emphasized the importance of research in implementing policies in the health sector, more importantly, in African middle- and low-income countries with high inequality in healthcare services due to poverty and lack of resources [7,8,9]. The current study was conducted in South Africa—a developing country where primary healthcare (PHC) encounters pivotal difficulties that have prompted patients to encounter negative encounters with healthcare, compromising the significant role that PHC services could play in health promotion and disease prevention [10].

In South Africa (SA), the IC program was initiated in July 2013 to improve the quality of PHC facilities. The IC has work streams with different building blocks capable of rendering excellent health services. The workstreams include how the institutional structure is arranged regarding the waiting period for patients, appointment dates, availability of human resource materials and supplies, and financial and administrative management [11]. More importantly, IC provides community-based health promotion and disease prevention programs in cooperation with the community. The IC aims to reduce long waiting period complaints and improve primary healthcare services, which the community members will be proud to use [11].

Additionally, the IC was designed to prevent a lack of quality patient care in PHC services. According to Fryatt and Hunter, South Africans are using clinics in increasing numbers, rising from 67 million in 1998 to 128 million in 2013 [6]. The achievements in PHC in SA over the past 20 years include improving immunization coverage for children younger than 1 year to nearly 100% and supporting more than 2.8 million patients on antiretroviral medication (ARV’S) [6]. Despite these achievements, Chandran, Tucker, and Ravhengani stated that PHC in SA faces serious challenges that have led to negative patient care experiences [12]. Research studies have identified the practice gap as a significant challenge in the healthcare system [13,14]. Several factors such as communication, adaptation, implantation, and evaluating different programs and guidelines were identified as the root cause of the practice gap in the healthcare setting [13]. In the context of this study, exploring the lived experiences of professional nurses will identify the gaps in implementing the IC.

The IC target provides a community-based, comprehensive range of integrated diagnostic, acute, preventative, rehabilitative, and chronic services [15]. The IC initiative’s implementation had its roots in the National Department of Health audit in 2011. The audit showed that public health facilities in SA scored less than 50% compliance with necessary measures, 34% in patient safety and security, and 30% in the area of positive and caring attitude [13]. Hospitals scored more than PHC in priority areas. The audit showed that PHC facilities are not providing a complete service. Essential drug supply was unreliable, staffing was inadequate, and the quality of infrastructure was affected. Research in Norway outlined a need to improve the provision and utilization of resources to improve and strengthen patient care and meet the patients’ different needs [15].

Meanwhile, the IC initiative was initiated to satisfy SA communities’ needs [6]. Although the IC program was designed to systematically transform all PHC facilities to meet national standards in preparation for the introduction of NHI, there seem to be challenges experienced with its implementation. The study aims to determine professional nurses’ experiences regarding implementing the IC at selected PHC facilities in Limpopo Province, SA.

## 2. Materials and Methods

This study adopted a qualitative phenomenological design to explore and gain more understanding through a personal and participant expression of the lived experience of professional nurses regarding the implementation of the IC program. Through phenomenological design, the professional nurses working in the selected clinics were able to express their experiences about implementing the IC as subjectively lived. Moreover, this enabled the researchers to discover the new meanings and appreciations that can be developed to inform or reorient the IC implementation [16,17].

### 2.1. Study Site

The study was conducted at three selected PHC facilities of Makhado Local Municipality. Makhado Local Municipality is located in Vhembe District, Limpopo Province. It is one of four municipalities in the district, comprising almost one-third of its geographical area. The Makhado Local Municipality is a predominately rural area with more than 200 villages and PHC facilities. Primary healthcare facility A delivers services to 4500 community members from 11 local villages monthly with 11 professional nurses who implement the IC. Primary healthcare facility B provides services to an average of 3500 community members from seven villages with 17 professional nurses. In comparison, clinic C delivers services to 2500 community members from nine villages with six professional nurses.

### 2.2. Population and Sampling

The population consisted of 34 professional nurses working in the three selected primary healthcare facilities in Makhado Local Municipality. A total purposive sampling strategy was used to determine professional nurses implementing the IC program on the basis of their knowledge and experience [17]. The sample size was guided by data saturation, which was reached at participant number 15.

### 2.3. Data Collection

Data were collected at the three selected PHC facilities through semi-structured open-ended one-on-one interviews. According to Jacobs and Furgerson, an open-ended inquiry exposes the human part of a story about participants’ experiences [18]. The participants were recruited through the assistance of the operational managers. The authors explained the study’s aim, possible benefits, and the voluntary nature of participation in the study to the professional nurses at the three selected primary healthcare facilities. Semi-structured in-depth interviews with an interview guide were conducted in a private room free of noise and distraction to facilitate free-flowing communication. One central opening question was asked: “Could you kindly describe your experience regarding the implementation of an IC program at your clinic?” Probing questions were asked to gather information and encourage the professional nurses to elaborate on the topic [19]. Since the authors aimed for a quality data analysis, a voice recorder was used to capture all the interview sessions. The interview sessions were conducted until data saturation was reached with participant number 15. The primary researcher suspended the presuppositions, biases, assumptions, theories, and previous experiences by writing all preconceived ideas before beginning with data collection [20].

### 2.4. Data Analysis

As described by Vaismoradi, Turunen, and Bondas, a thematic descriptive approach was applied to analyze data by examining descriptions of lived experiences [21]. The primary researcher familiarized herself with data by reading all the verbatim transcriptions, carefully writing down the initial ideas. Initial codes were generated by systematically coding interesting features and organizing data to each relevant code. Themes and subthemes were developed from coded data and the associated texts (see Table 1). The final analysis report was produced selecting extracts of the lived experiences of the professional nurses, narrating back to the research question and the study objectives [21].

### 2.5. Trustworthiness

Trustworthiness was ensured by adhering to Guba’s four criteria: credibility, dependability, confirmability, and transferability. We ensured credibility by recording all the interview sessions and capturing the field notes as proof of the participants’ data collection. Transferability was ensured by the detailed description of the research methodology. Dependability was ensured by keeping safe all the voice recordings and field notes. To ensure confirmability, the researchers submitted the verbatim transcripts to the independent coder, and a meeting was set to discuss the themes and subthemes, which were reached independently [22].

## 3. Results

The findings are presented in themes and subthemes (see Table 1), supported by narratives, which are the participants’ direct quotations, written in italics.

### 3.1. Theme 1: Perceived Benefits of the IC Program on the PHC Services Provided to the Community

Professional nurses perceived the IC as a program that benefits and improves the standard of the primary healthcare service. The benefits include, firstly, improved access to PHC services to the community members through facility reorganization. Facility reorganization is when the PHC facility is reorganized to eliminate congestion in the facility [23]. Additionally, in an organized facility, the patients are aware of movement channels from the gate when they enter the clinic until the consulting area. Secondly, there was a reduction in workload and prolonged waiting periods for communities to receive PHC services. The “Operation Phakisa” IC Realization and Maintenance (ICRM) laboratory developed the planning for implementing the IC. It recommended that patients’ maximum waiting time be 3 h, and that supplies and medicines should be available.

### 3.2. Theme 2: Challenges Experienced by Professional Nurses When Implementing IC Program

Professional nurses reported that, despite the benefits experienced, there were challenges during the IC implementation. These challenges were explained according to subthemes. Firstly, professional nurses expressed concerns regarding the maintenance of equipment for the effective implementation of the IC program. The maintenance of equipment, as well as the replacement of broken furniture and machinery, affected the implementation of the IC program. Secondly, professional nurses expressed concerns regarding an IC program that was implemented abruptly. Professional nurses implementing the IC program need to be trained and tested on their knowledge regarding the program’s content. Training should be done through long- and short-term courses on the implementation of an IC program, thus indicating the significance of the in-service training that could be conducted at PHC facilities to orientate all professional nurses [12]. Lastly, a lack of involvement of the professional nurses in implementing the IC program resulted in a negative attitude and behavior. Practical orientation should enforce positive behavior with the implementation of the IC program. Orientation should make the professional nurses feel more secure, leading to the IC program’s enjoyment. The orientation program could result in more caring for the patients, ultimately seeing the benefits and loving the program’s implementation. Professional nurses need to be observed by their peers to pre-empt insecurities when critiquing the implementation of an IC as it requires creativity. The study results revealed that, initially, the participants were afraid of the changes brought by the new Ideal Clinic program.

### 3.3. Theme 3: Challenges Regarding the Supply of Resources for the Implementation of the Ideal Clinic

Participants expressed concern with the provision of essential drugs and other resources to implement the IC. Two subthemes emerged. Firstly, professional nurses pledged that patients’ actual help would be their priority; however, with an inadequate supply of essential drugs during emergency care services, this became a dream. With the IC program, priority became the infrastructure, administration, and clinic cleanliness, causing further challenges with the supply of drugs. Secondly, there was a lack of provision of human and other resources to implement the IC program. When the IC program was introduced, there were some guidelines and specifications on how it should be implemented; however, participants reported that the resources for implementation were not provided.

## 4. Discussion

Despite the global commitment to strengthen the PHC systems as an essential step toward achieving universal health coverage, the actual implementation has not exceeded expectations [24]. This study aimed to determine professional nurses’ experiences regarding implementing the IC program at three primary healthcare facilities in Vhembe District, Limpopo Province, SA. The findings highlighted a mismatch between professional nurses’ experiences and the expected improvements of the IC program. One of the prescribed roles of the professional nurses in the PHC facilities outlined by the South African Nursing Council (SANC) is quality improvement through the development and implementation of clinical protocols and guidelines related to the area of practice [24]. However, in this study, the professional nurses experienced challenges in implementing the IC, which is one of the protocols designed to improve quality in the PHC services.

This study revealed the good experiences of professional nurses regarding the implementation of the IC program as one of the programs which brought changes and benefits to the PHC system. These benefits included the reorganization of PHC facilities and integration of services, wherein the patients are managed in the same centralization of care. Additionally, with the IC program, the facilities were reorganized from the clinic entrance, whereby there is signage to guide the patients to the designated waiting area, and all the consulting rooms are labeled. These results concur with the literature IC program manual, which specifies that the facility must be reorganized with a designated consulting area and staffing for acute, chronic health conditions and preventative health services [25]. The manual further states that process flow mapping should be achieved by drawing up the facility’s floor plan [25]. The floors should be marked using color coding to direct patients’ paths with three different colors, e.g., acute using orange or red, chronic using blue, and preventative using green.

Professional nurses’ experiences revealed changes brought by the implementation of the IC program, such as changes in the booking system for all patients with chronic diseases and those seeking preventative services, as well as a reduction in workload. Professional nurses expressed that the booking system changes helped reduce the workload in the facility because patients came per appointment. In support of the results, the IC manual outlined that the booking system should operate as a function of date and time for patients to come and collect their treatment [25]. The facility must be reorganised with a designated consulting area and staffing for acute, chronic health conditions, and preventative health services. Furthermore, all planned care streams should be efficiently organized and properly managed through a proper patient appointment system for patients with stabilized chronic health conditions [26]. In contrast, the changes brought by the Ideal Clinic were viewed by some participants as a challenge to increase the workload of the staff members due to centralized care where all the services were provided to patients at the same time.

The study findings revealed that professional nurses experienced challenges regarding implementing the IC program since the program requires early identification, reporting, and recording of broken equipment. Professional nurses expressed concerns regarding delays in reporting broken equipment and machines, as well as the long period taken to have them fixed. This finding concurs with the study by Kredo, Cooper, Abrams, et al., conducted in four provinces in South Africa, which suggested an urgent need for the National Department of Health (NDoH) to prioritize the existing challenges in the healthcare system in accessing and using medical supplies, which impacted adherence to guidelines [27]. Steinhobel, Mossyn, and Peer outlined that the implementation of the IC program includes maintenance of the infrastructure and the availability of resources [28]. Furthermore, maintenance is about keeping the workplace, structure, equipment, furniture, and facility in good condition.

Professional nurses expressed concerns regarding an IC program that was implemented abruptly with unclear descriptions of professional nurses’ roles [25]. The participants described a negative experience of the program related to the knowledge gap related to a lack of training in implementing the IC program as some of the experiences they faced. They further explained that they were only told to follow the line managers’ IC manual and indicators when the program started. This resulted in poor performance and a lack of interest in the program because each one understood the manual. The clinical service provision element of the IC program states that there should be in-service training for all staff on integrated clinical services management [25]. Orientation gives an understanding of how, where, when, what, who, and with whom to implement an IC program as it is a new challenge [12]. Some participants revealed that they lacked knowledge and insight into the IC program, while the other professional nurses described unclear roles to play during the program. The only person with knowledge of implementation was the operational manager who gained guidance from the South Africa manual documents of IC components regarding the program’s implementation, including administration and integrated clinical services management (ICSM), with sub-elements to show commitment for IC implementation [26].

In line with the existing study by Kredo, Cooper, Abrams, et al., a lack of professional nurses’ involvement (who are the end-users in implementing the IC program) was revealed, which resulted in a lack of interest and negative attitude and behavior [27]. Different researchers also raised a concern that lack of consultation and communication with the end-users of a policy has a negative impact on the implementation of such a policy [29,30,31]. Other participants described having to use the old way to implement the IC because they were not oriented. This finding is not in line with the IC manual, which states that professional nurses must be orientated to the Ideal Clinic program, which would allow them to enjoy and be committed to implementing the IC program [25]. The literature states that nurses need to undergo specific training and workshops to improve their knowledge and manage the patient properly [28]. Practical orientation enforces positive behavior in the implementation of the guidelines.

Challenges related to the availability of treatment and resources were reported as barriers to effectively implementing the IC clinic. Such challenges were inadequate supply of essential drugs during emergency care services, shortage of stationery, and staff shortage. The program has different streams, whereby, in each consulting room, there should be a professional nurse. This was one of the participants’ significant concerns as there was inadequate staffing to achieve this. This finding concurs with Muthathi, Laetitia, and Rispel’s study that revealed a shortage of human resources and equipment as a major challenge. It is recommended for the Department of Health, SA, to provide healthcare workers and adequate resources in the PHC facilities to properly implement the IC program [31].

The IC program in SA was introduced into PHC facilities as part of the preparation for the implementation of National Health Insurance (NHI). The NHI aims to ensure that universal healthcare for all in South Africa is provided. On the other hand, the IC program aims to improve the quality of patient care.

The current study suggests that the implementation of the IC program remains a challenge in the PHC facilities; thus, the provision of quality patient care cannot be achieved. The study recommends that, to improve the identified gaps in the implementation of the IC program, the National Department of Health (NDoH) in SA should consult with the end-users before and during the implementation to improve the quality of patient care. It is worth mentioning that professional nurses can effectively implement the IC program if they are well trained, continually supported, and provided with adequate staff, supplies, and quality equipment.

### Limitation of the Study

This study was conducted at only three PHC facilities in the Makhado Local Area of the Vhembe District. Therefore, the results of the study cannot be generalized to other local areas. The IC program is still new and has only been implemented in South Africa and Malaysia; therefore, data from international and African studies are limited.

## 5. Conclusions

According to the study results and the description of how the IC should be implemented, “the IC is a clinic with good infrastructure, adequate staff, adequate medicine and supplies, good administrative processes, and sufficient bulk supplies, which uses applicable clinical policies, protocols, guidelines, and partner and stakeholder support to ensure the provision of quality health services to the community” [25]. The study, therefore, concludes that, according to the lived experiences of professional nurses, there are benefits and challenges to the implementation of IC. Benefits include improved access to primary healthcare services and a reduction in workload. In contrast, the challenges toward the successful and effective implementation of the IC in three PHC facilities of the Makhado Local Area include poor implementation of the IC, as well as a lack of resources, essential drugs, and equipment maintenance.

## Figures and Tables

**Table 1:** Summary of themes and subthemes that emerged from data analysis reflecting professional nurses’ experiences regarding the implementation of the Ideal Clinic program.

Themes	Sub-Themes	Count (*n* = 15)
1. Perceived benefits of the Ideal Clinic on the primary healthcare services provided to the community	1.1. Improved access to primary healthcare services by the community through facility reorganization	3
	1.2 Changes in the booking system with reduction of workload and prolonged waiting periods for communities to receive primary healthcare services	4
2. Challenges experienced by professional nurses when implementing the IC program	2.1 Professional nurses expressed concerns regarding the maintenance of equipment for the IC program	4
	2.2 Professional nurses expressed concerns about an IC program that was implemented abruptly	6
	2.3 Lack of involvement of the professional nurses with implementing the IC program resulted in a negative attitude and behavior	6
3. Challenges regarding the supply of resources for the implementation of the IC program	3.1. Inadequate supply of essential drugs during emergency care services	4
	3.2. Lack of provision of human and other resources to implement the IC program	3

## Data Availability

Data generated and analyzed during the current study are not publicly available due to ethical reasons but are available from the corresponding authors.
